# Multi-Band Power Amplifier Module with Back-Off Efficiency Improvement using Ultra-Compact 3D Vertical Stack Multi-Chip Package for Cellular Handsets

**DOI:** 10.3390/mi13111976

**Published:** 2022-11-15

**Authors:** Zhihao Zhang, Jing Li, Lin Peng, Bo Sun

**Affiliations:** 1 School of Integrated Circuits, Guangdong University of Technology, Guangzhou 510006, China; 2 Institute of Public Policy, South China University of Technology, Guangzhou 510610, China

**Keywords:** power amplifier module (PAM), multi-mode multi-band, high efficiency, 3D vertical stack, hybrid bulk CMOS/GaAs HBT/SOI, miniaturization

## Abstract

A highly integrated multi-mode multi-band (MMMB) power amplifier module (PAM) using hybrid bulk complementary metal oxide semiconductor (CMOS), gallium arsenide (GaAs) heterojunction bipolar transistor (HBT), and silicon-on-insulator (SOI) technologies for low band (LB, 824–915 MHz) and high band (HB, 1710–1980 MHz) is proposed. The hybrid MMMB PAM integrates a bulk CMOS controller die, a GaAs HBT power amplifier (PA) die and a SOI switch die on a six-layer laminate. To simultaneously obtain both highly efficient and highly linear characteristics over a wide range of input power levels, a parallel dual-chain PA strategy has been adopted to provide vary bias current and gain for low-power mode (LPM) and high-power mode (HPM) operation. Additionally, a broadband two-section low-pass output matching network design based on the suppression of high-order harmonics is proposed for enhanced efficiency and linearity. In order to achieve further miniaturization, a three-dimensional (3D) die stack multi-chip module (MCM) packaging structure, where the presented CMOS controller die is stacked vertically on the GaAs HBT PA die, is implemented. The measurement results show that the fabricated MMMB PAM achieves 26.1–27 dB of power gains and 38–38.4% of PAEs at an output power (Pout) of 28 dBm in the HPM, and 20.4–20.9 dB of power gains and 12.4–13.8% of PAEs at Pout of 17 dBm in the LPM over LB. For HB, power gains of 24.3–26.7 dB while maintaining PAEs of 38.2–39.9% at Pout of 28 dBm, and power gains of 15.9–17.5 dB while maintaining PAEs of 12.3–12.8% at Pout of 17 dBm are realized in the HPM and LPM, respectively. The fabricated PAM covering five frequency bands and operating at two power modes only occupies a 5 × 3.5 mm^2^ area. To the best of the authors’ knowledge, this work is the first demonstration of a MMMB PAM adopting an ultra-compact 3D vertical stack MCM package with favorable RF performance.

## 1. Introduction

As more and more mobile communication standards (2G/3G/4G/5G) are incorporated in modern cellular handsets, single-band power amplifiers (PA) are not suitable anymore, which places multi-mode multi-band (MMMB) power amplifier modules (PAMs) in an increasingly vital role in cramping multiple bands into a single radio frequency (RF) front end to support the growing number of frequency bands for mobile device applications [1,2,3].

Generally, a complete MMMB PAM consists of one PA controller for diverse control levels, one or more PA blocks operating in multiple bands and one post-PA band-selected switch to separate different bands. A common perception is that bulk silicon complementary metal oxide semiconductor (CMOS) is currently an extremely suitable integrated circuit technology for high integration and low cost [4]. Thus, for the PA controller design, bulk CMOS is a cost-effective solution due to its ability to incorporate a massive number of transistors on a single die. However, in the fields of the PA blocks, as the modulation complexity and data rates increase in cellular communication, the high linearity and high peak-to-average ratio requirements have limited the use of the bulk CMOS process due to its inherently low power density and conductive substrate characteristics. To realize acceptable RF performance, most of the currently available PAs for handsets employ gallium arsenide (GaAs) heterojunction bipolar transistor (HBT) technology since it reveals higher power density and better linearity characteristic over the bulk CMOS device in high frequency [5,6,7]. Additionally, silicon-on-insulator (SOI) CMOS technology is more desirable compared to the bulk CMOS in the RF switch design thanks to its insulating substrate that lowers substrate losses and parasitic capacitances, and most importantly, compatibility that is able to integrate the analogy circuitry [8,9]. All things considered, in this article, we have implemented a handset MMMB PAM in hybrid bulk CMOS/GaAs HBT/SOI technologies, where a bulk CMOS process is used for the PA controller design, a GaAs HBT technology is utilized for the design of PA blocks and a SOI process is utilized for the post-PA band-selected switch design.

Commercially cellular wideband PAs are required to operate with high efficiency with high output power back-off regions. However, conventional PAs are designed to operate with significant back-off for high linearity, thereby remarkably decreasing the power efficiency [10]. To accommodate a higher data rate and extend battery life, many strategies for efficiency improvement over a wide power range, i.e., envelope tracking (ET) [11,12], envelope elimination and restoration (EER) [13], Doherty [14,15] and adaptive load [16,17]/bias [18,19] for high-efficiency linear PA have been investigated, but the complexity and cost are critical drawbacks. Moreover, various output matching topologies have been developed to meet the tough efficiency requirements at linear output power levels across a broadband frequency range. By squaring the output voltage waveform via the output network based on lumped-element resonators, Class-F/F^−1^ [20,21] operation can be achieved to provide excellent efficiency. Additionally, assisted by the capacitive second-order harmonic frequency impedance, Class-J/J^−1^ [22,23] loading networks exhibit outstanding efficiency performance, but their complexity and cumbersomeness are major obstacles for high-integration PAMs. In addition, it had been demonstrated that controlling second- or higher-order harmonics helps to deliver high efficiency in the PA design [24,25,26]. In this work, to improve the power added efficiency (PAE) at power back-off while achieving multiband operation, a parallel dual-chain two-stage PA strategy for different power modes is realized and a broadband output matching network (OMN) design based on the suppression of high-order harmonics is proposed.

In addition to the requirement of multiband operation with higher efficiency, multiband operation with smaller size has lately been strongly expected for cellular PAMs. Currently, a smart mobile device is capable of easily operating in more than 20 cellular bands and other non-cellular wireless services, revealing the complexity inherent with multiple RF chips operating and coexisting within a small physical volume. Thus, the implementation of handset PAMs with small size has become extremely crucial. Although the use of MMMB PAMs is highly beneficial to reduce the number of PAs and save size area, most PAMs reported or released using traditional planar multi-chip module (MCM) packaging structures still occupy large chip sizes [27,28,29,30]. Consequently, to achieve further miniaturization, a novel three-dimensional (3D) die stack packaging structure instead of a planar structure is adopted in our proposed PAMs, in which a presented bulk CMOS controller die is stacked vertically on a GaAs HBT PA die to realize the requirement of size reduction. To the best of the authors’ knowledge, this work is the first demonstration of a MMMB PAM using a 3D vertical stack MCM package with superior RF performance for mobile handset applications.

## 2. Circuit Design

### 2.1. MMMB PAM Architecture

**Figure 1:**
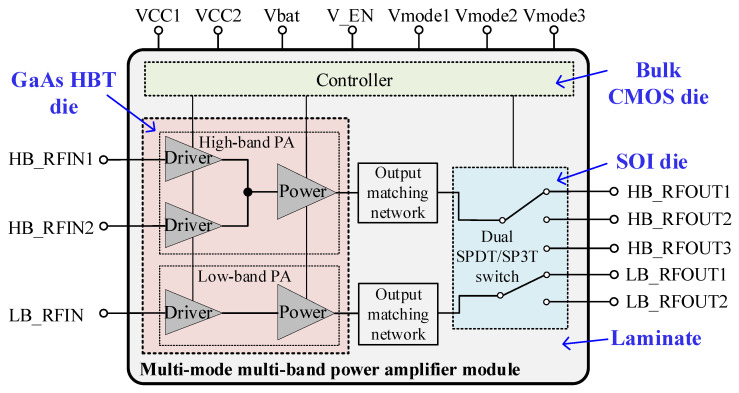
Simplified block diagram of proposed multi-mode multi-band (MMMB) power amplifier module (PAM).

Figure 1 describes the simplified block diagram of the proposed MMMB PAM, which integrates a GaAs HBT PA die, a SOI dual single-pole double-throw (SPDT)/single-pole triple-throw (SP3T) switch die, a bulk CMOS controller die and multiple passive components mounting on a multi-layer laminate substrate. The PAM adopts an improved architecture to support penta-band cellular modulations. There are two PA blocks inside the GaAs HBT die for the LB (824–915 MHz) and HB (1710–1980 MHz). Both LB and HB PA blocks utilize a two-stage solution and are composed of a driver stage and a power stage. Particularly, in contrast to the LB PA block, the HB PA block has two switchable driver stages to separate the input signal.

### 2.2. GaAs HBT PA Design

#### 2.2.1. Two-Stage Dual-Chain HBT PA Configuration

**Figure 2:**
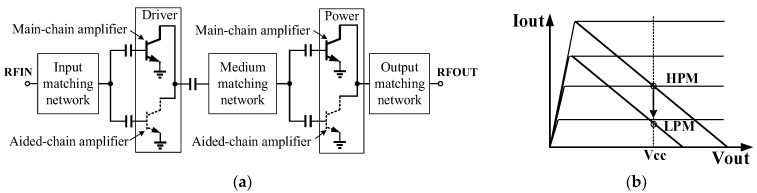
Two-stage dual-chain heterojunction bipolar transistor (HBT) PA configuration: (**a**) parallel dual-chain PA strategy with common matching networks; (**b**) load line characteristic for high-power mode (HPM) and low-power mode (LPM).

Typically, the efficiency of a PA decreases as the input power level decreases. At a small power level, the operating points of a PA are lowered further away from its saturation point, leading to severe PAE degradation [31,32]. In this work, to simultaneously achieve both high efficiency and high linearity over a wide power range, a parallel two-stage dual-chain strategy with a common input, medium and output matching network, illustrated in Figure 2a, have been adopted to provide vary bias current and gain for low-power mode (LPM) and high-power mode (HPM) operation. In the HPM, both the main-chain and aided-chain amplifiers in the driver stage and power stage are activated with a high quiescent operating point for a high gain, and a 1 dB compression point (P1dB) of 28 dBm can be achieved. On the other hand, only the main-chain amplifiers are activated and the aided-chain amplifiers in both driver and power stage are inactivated in the LPM, leading to the drop down of the DC bias current resulted from the decrease in the emitter area, as depicted in Figure 2b. Under the circumstances, the PA exhibits a low power gain and low P1dB of 17 dBm, thus benefitting the efficiency improvement in the presence of a low input power level. More importantly, the proposed two-stage dual-chain HBT PA strategy only adopts a single common input, medium and output matching networks. Extra matching networks or switching circuits that might degrade the performance and increase the chip size are not required.

#### 2.2.2. Broadband Output Matching Technique

**Figure 3:**
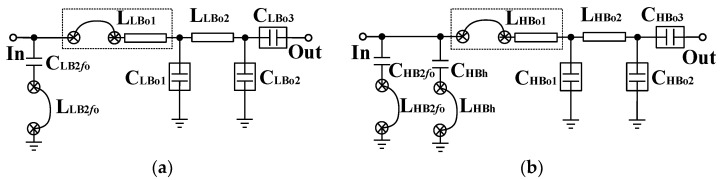
Output matching network for: (**a**) low band (LB); (**b**) high band (HB).

**Figure 4:**
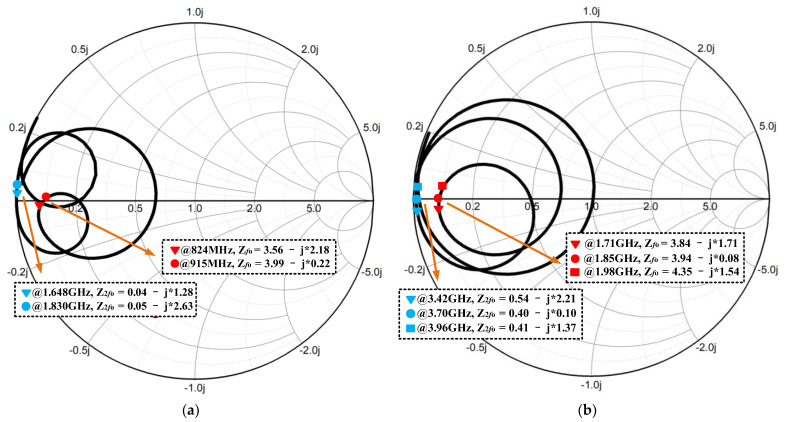
Optimum load impedance traces for: (**a**) LB; (**b**) HB.

The design of the common input, inter-stage and output impendence matching networks are of great importance due to their sensitivities to frequency and RF performance [33]. In particular, the OMN design is most essential because it determines the bandwidth, output power level and efficiency [34,35]. This work presents a novel broadband two-section low-pass matching technique, as shown in Figure 3, which is designed to embed high-order harmonic manipulations for realizing harmonic suppression and efficiency promotion for the LB and HB PA blocks. In Figure 3a, the LB output matching circuit mainly contains 9 elements. At the fundamental frequency (*f*_0_), L_LBo1_, C_LBo1_, L_LBo2_ and C_LBo2_ form a second-order LC low-pass matching network, which plays the role of transforming the 50 Ω to the optimum load impedance (Ropt ≈ 3.5 Ω) at *f*_0_. The C_LBo3_ is a DC blocking capacitor. The C_LB2*f*0_ and L_LB2*f*0_ form the second-order harmonic frequency (2*f*_0_) trap, so that the output network can obtain a short-circuit load at 2*f*_0_ for realizing Class-F operation. The voltage waveform at the collector of the power-stage transistor exhibits sharper edges than sinusoid, lessening the overlay time between the voltage across and the current flowing in the transistor, thereby reducing the power loss. Similar to the LB, the HB output matching circuit is shown in Figure 3b. Considering the fact that higher harmonics can be exploited to further minimize the time during which the transistor maintains a large voltage and carries a large current, an additional tank consisting of C_HBh_ and L_HBh_ is added in the HB output matching network and is optimally designed between twice and three times the fundamental frequency to further realize different termination impedances for different harmonics, thereby improving the efficiency while sustaining good linearity. Figure 4a,b show the optimum load impedance traces on Smith Charts for both LB and HB.

L_LBo1_, L_LB2*f*0_, L_HBo1_, L_HB2*f*0_ and L_HBh_ in the output matching circuits are composed of bond-wire inductance and laminate inductance. L_LBo2_ and L_HBo2_ are laminate inductances, and L_LBo3,_ L_LBo4_, L_HBo3_ and L_HBo4_ are bond-wire inductances. The C_LB2*f*0,_ C_HB2*f*0_ and C_HBh_ with moderate capacitances, are placed on the HBT die. Six surface-mount devices (SMDs), C_LBo1_, C_LBo2_, C_LBo3_, C_HBo1_, C_HBo2_ and C_HBo3_, are used on the laminate to reduce the HBT die size and obtain low loss.

#### 2.2.3. Detailed Structure of Proposed MMMB PA

**Figure 5:**
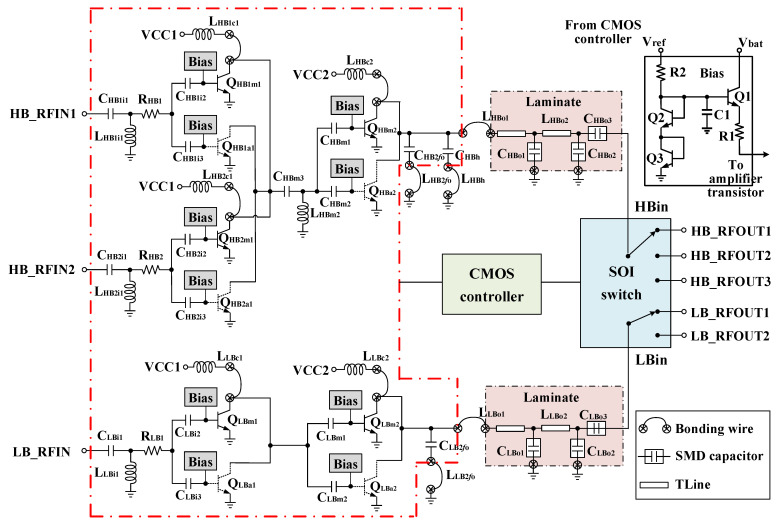
Detailed structure of proposed MMMB PA.

Figure 5 depicts the detailed schematic of the proposed two-stage dual-chain MMMB PAs for both LB and HB. In the LB PA block, a same emitter area of 504 um^2^ has been employed for both main- and aided-chain amplifiers (Q_LBm1_ and Q_LBa1_) in the driver stage and an identical emitter area of 3570 um^2^ has been adopted for the dual-chain amplifiers in the power stage (Q_LBm2_ and Q_LBa2_). In the HB block, a two-stage strategy with a switchable driver stage is presented for multiband operation. The inputs of the first and the second driver stages are connected to HB_RFIN1 and HB_RFIN2, respectively, and the outputs of both of them are connected to the input of the second power stage. The main- and aided-chain amplifiers in the first driver stage (Q_HB1m1_ and Q_HB1a1_) have the same emitter area of 336 um^2^, while for the second driver stage, (Q_HB2m1_ and Q_HB2a1_) have the same emitter area of 378 um^2^ and the dual-chain amplifiers in the power stage (Q_HBm2_ and Q_HBa2_) have an identical emitter area of 1932 um^2^. In the HPM, a relatively high quiescent current of approximately 60–70 mA is shown as both the dual-chain amplifiers in the driver stage and power stage of both LB and HB PA blocks are activated. In the LPM, only the main-chain amplifiers are enabled and the aided-chain amplifiers in both driver and power stage of the LB and HB blocks are disabled, leading to a low quiescent current of approximately 30 mA.

In view of the trade-off between efficiency and linearity, the proposed two-stage dual-chain PAs in both LB and HB have Mid-Class AB operation for the driver stages and Deep-Class AB operation for the power stages. However, the second-stage PA operating with a low quiescent bias point and high-efficiency output matching design would lead to gain expansion and lag phase shift, which in turn introduces severe nonlinear distortion and reduces linearity. To compensate for the distortion, the first-stage PA is designed to realize gain compression and lead phase shift by using appropriate matching networks and higher quiescent current bias. As a result, the first and second stage complementarily cancel the nonlinear components of AM–AM and AM–PM, thereby obtaining relatively flat gain and phase characteristics and improving linearity.

A detailed description of the broadband OMN design is illustrated in Section 2.2.2. The inter-stage matching network (ISMN) of the proposed work is carefully designed on chip to transform the input impedance of the second stage to the optimal load impedance of the first stage for achieving sufficient gain and output power capability. It can be observed in Figure 5 that a single-section L-type matching structure including L_LBc1_ and C_LBm1_/C_LBm2_ is used for the ISMN of the LB PA block, whereas the HB PA block adopts a two-section L-type matching structure including L_HB1c1_/L_HB2c1_, C_HBm3_, L_HBm2_ and C_HBm1_/C_HBm2_. The L_LBc1_, L_HB1c1_ and L_HB2c1_ consisting of bond-wire inductance and laminate inductance are also used to feed DC voltage to the first-stage amplifier. The conjugate match design is chosen for the input matching network (IMN) to realize maximum gain and minimum reflection loss. The IMN for both LB and HB blocks is designed using a simple L-type matching structure involving a series capacitor (C_LBi1_/C_HB1i1_/C_HB2i1_) and a shunt inductor (L_LBi1_/L_HB1i1_/L_HB2i1_). To improve the stability, a small series resistor of 5-Ohm (R_LB1_) and 8-Ohm (R_HB1_ and R_HB2_) for LB and HB PA blocks, respectively, is added before the base of the first-stage transistor. The reason we did not employ the stabilized resistor in the second stage is due to its significant deterioration of efficiency and linearity. Eventually, linear bias circuits are employed to the overall circuit.

### 2.3. SOI Switch Design

**Figure 6:**
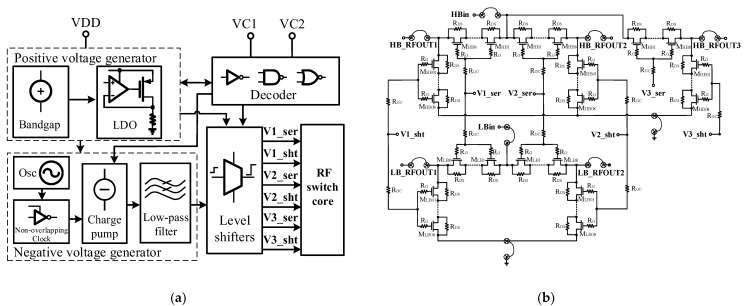
Silicon-on-insulator (SOI) dual single-pole double-throw (SPDT)/single-pole triple-throw (SP3T) switch: (**a**) block diagram; (**b**) detailed structure of RF switch core.

Figure 6a illustrates the 0.18 μm SOI switch controller structure, where the voltage regulator consisting of a bandgap and low dropout regulator (LDO) is used to provide a stable positive voltage of +2.5 V, the charge pump is employed to generate a negative voltage of −2.5 V and the level shifters are utilized to switch the voltage level from +2.5/0 V to +2.5/−2.5 V for the gate bias of the switch-FETs in the on and off states, respectively. Figure 6b shows the detailed RF-core configuration of the presented SOI switch, which comprises a SPDT configuration for LB and a SP3T configuration for HB. The saturated output power of the PA is better than 28 dBm. In order not to limit the linearity of the PA, eight stacking transistors are employed for both series and shunt arms to handle a power level up to 31 dBm with a voltage standing wave ratio (VSWR) as high as 5:1. The device peripheries in series and shunt arms are set to 3000 and 500 um, respectively, with the given stack height to support the IL and isolation requirements. Switching is controlled by two logic voltages, VC1 and VC2, from the bulk CMOS controller. Depending on the VDD and logic voltage level, the LBin signal is connected to one of two switched RF outputs (LB_RFOUT1 or LB_RFOUT2), while the HBin signal is simultaneously switched to one of three RF outputs (HB_RFOUT1, HB_RFOUT2 or HB_RFOUT3).

### 2.4. Bulk CMOS Controller Design

**Figure 7:**
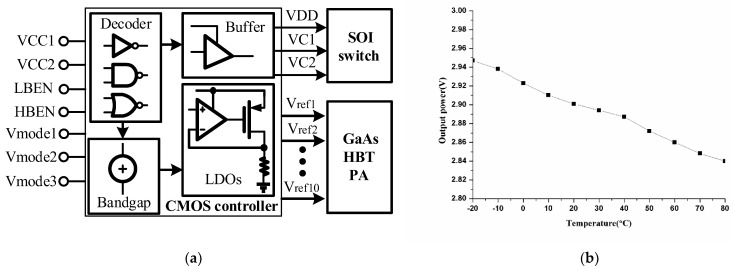
Proposed bulk CMOS controller: (**a**) block diagram; (**b**) temperature drift curse of output power.

To ensure that the power gain of the power amplifier is constant under different ambient temperatures, different transmit powers and different operating voltages, the output voltage of the PA controller is required to have a deviation of less than 50 mV at room temperature. Figure 7a reveals the proposed controller structure based on a 0.18 μm bulk CMOS technology, which consists of a logic circuitry, a bandgap reference source (BGR) and multiple low dropout linear regulators (LDOs) and a buffer. The logic circuitry converts the logic control signal to the internal supply voltage and decodes it into the control signal for the subsequent BGR and the SOI switch through a buffer. The BGR is used to provide voltage reference and current bias for the subsequent LDOs. The LDOs aim to offer suitable bias regular voltages for the HBT PA. Since the CMOS controller is required to provide voltage biases with a specific temperature coefficient for the HBT PA, the output voltages of the CMOS controller are designed to have a negative temperature coefficient to compensate for the negative temperature coefficient of the triode base-emitter voltage. Figure 7b plots the temperature drift curse of Vref1 (one of the controller output voltages). It can be calculated that the average voltage is 2.89 V and the temperature coefficient is approximately −370 ppm/°C.

### 2.5. Laminate Design

**Figure 8:**
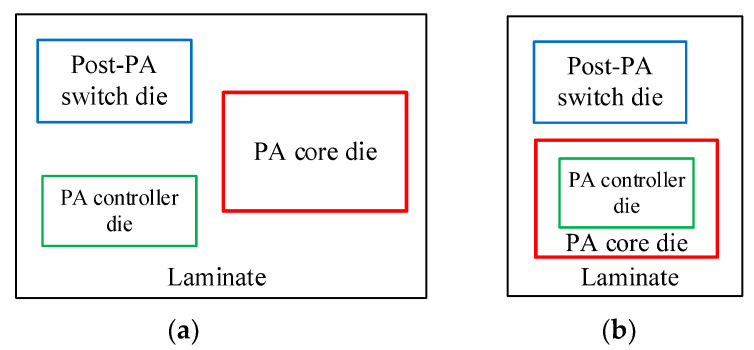
Multi-chip module (MCM) package structure for MMMB PAM: (**a**) conventional planner package; (**b**) three-dimensional (3D) vertical stack package.

A MMMB PAM generally uses a MCM package structure where the laminate design is extremely essential as it contributes to the efficient interconnection of multiple dies and SMDs, thereby reducing assembling loss and achieving high performance. As shown in Figure 8a, a multiband PAM typically uses a conventional planer MCM packaging structure, where multi-functional dies are directly mounted on a laminate. To achieve smaller size and realize further miniaturization, a novel 3D die stack packaging structure instead of a planar structure is adopted in our proposed PAMs, as shown in Figure 8b, in which the PA controller die is stacked vertically on the PA-core die to realize size reduction.

**Figure 9:**
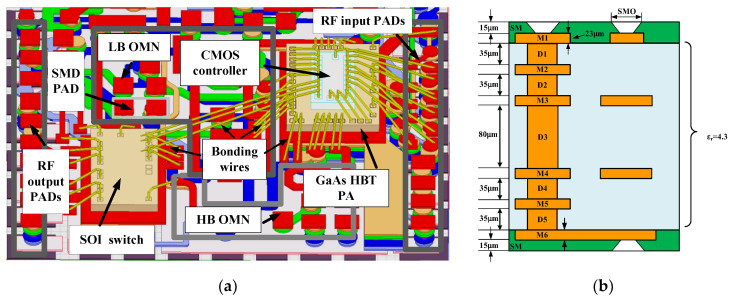
Proposed laminate design: (**a**) 3D view on which bulk CMOS/GaAs HBT/SOI dies assembled with bonding wires; (**b**) cross section of layer structure.

Figure 9a exhibits the laminate designed in this work. Note that the SOI die and the GaAs HBT die are mounted directly on the laminate, whereas the bulk CMOS die is 3D-stacked vertically on the GaAs HBT die to save physical volume. Golden bond wires are used to form electrical connections between the laminate and different dies. In particular, the transmission of regular voltage signals (V_ref1_, V_ref2_, …, V_ref10_) are directly realized via the bond wires between the CMOS die and the GaAs die. Similarly, the transmission of controller signals (VDD, VC1 and VC2) are directly implemented through the bond wires between the CMOS die and the SOI die. Additionally, as described in Figure 4 above, several matching transmission lines and capacitors (C_LBo1,_ C_LBo2,_ C_HBo1_ and C_HBo2_) of the LB and HB PA OMNs are designed on the laminate to achieve lower loss and better efficiency for broadband operation. As depicted in Figure 9b, the proposed laminate is based on a multilayered structure containing six copper metal layers (M1, M2, …, M6) with the same thickness of 23 um and five epoxy dielectric layers (D1, D2, …, D5) with the same relative dielectric constant (ε_r_) of 4.3. The thickness of the D3 layer is 80 um while the rest (D1, D2, D4 and D5) are of the same thickness of 30 um. To investigate all the parasitic coupling effects and enhance the design accuracy, the electromagnetic (EM) simulation for the proposed laminate, including the output matching elements and all RF signal bonding wires, is carried out.

## 3. Measurement Results and Discussion

**Figure 10:**
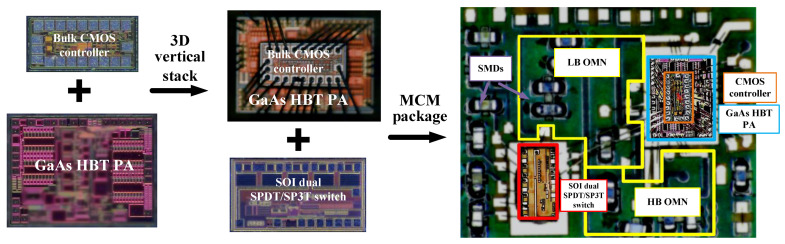
Microphotograph of proposed MMMB PAM with hybrid bulk CMOS/GaAs HBT/SOI technologies in 3D vertical stack MCM package.

Figure 10 shows the complete microphotograph of the proposed MMMB PAM that integrates the designed GaAs HBT PA die, SOI switch die and bulk CMOS controller die on the implemented laminate using a MCM package. To achieve further miniaturization, the CMOS controller die is 3D stacked vertically on the HBT PA die. Thus, an entire chip size as small as 5 × 3.5 mm^2^ is realized for the proposed PAM, where the die sizes of the HBT PA, SOI switch and CMOS controller are 1.45 × 1.05 mm^2^, 1.3 × 0.7 mm^2^ and 0.84 × 0.44 mm^2^, respectively.

**Figure 11:**
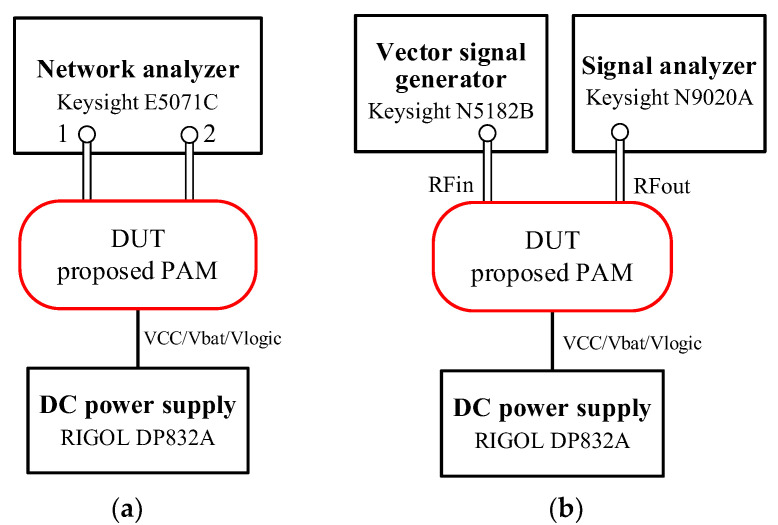
Measurement setup of proposed MMM PAM: (**a**) small-signal; (**b**) large-signal.

On-evaluation-board small-signal and large-signal measurements were carried out for the broadband PAM under 3.4 V of power supplies (VCC1, VCC2 and Vbat) and 1.8 V of control logics (V_EN, Vmode1, Vmode2 and Vmode3). Figure 11a depicts the small-signal S-parameter and stability factors (k and b) experiment setup using a Keysight E5071C network analyzer and RIGOL DP832A DC power supply, and Figure 11b illustrates the large-signal parameter measurement setup with a Keysight N5182B vector signal generator, Keysight N9020A signal analyzer and RIGOL DP832A DC power supply.

**Figure 12:**
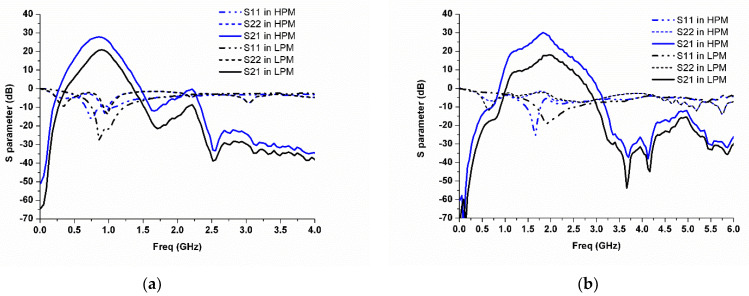
Measured small-signal S-parameters in the HPM and LPM: (**a**) LB; (**b**) HB.

Figure 12 shows the measured S-parameters of the proposed MMMB PA in both HPM and LPM for the LB and HB. It can be seen in Figure 12a that small-signal power gains (S21) varying from 27.5–27.8 dB and 19.7–20.9 dB are obtained in the HPM and LPM, respectively, over the frequency range from 824 to 915 MHz for the LB. In both modes, input return loss (IRL) of better than −9 dB are achieved. As shown in Figure 12b, the HB PA delivers S21 ranging from 28–30 dB and 16–19 dB in the HPM and LPM, respectively, with IRLs of better than −9 dB for 1710 to 1980 MHz frequency range. Additionally, it can be noted in the HPM that with the use of the proposed broadband output matching technique, the differences of output power between the fundamental and the second-/third-order harmonic frequencies are as large as approximately 45/60 dB and 60/60 dB for the LB and HB, respectively.

**Figure 13:**
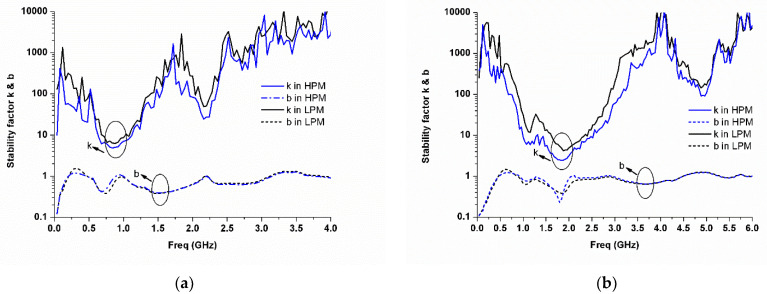
Measured stability k and b in the HPM and LPM: (**a**) LB; (**b**) HB.

Figure 13a,b reveal the measured stability factor k and b of the proposed MMMB PA in both HPM and LPM for the LB and HB, respectively. The k and b of greater than 1 and 0, respectively, are achieved over a wide frequency band of interest, which verified the proposed PAM is in an absolutely stable operation.

**Figure 14:**
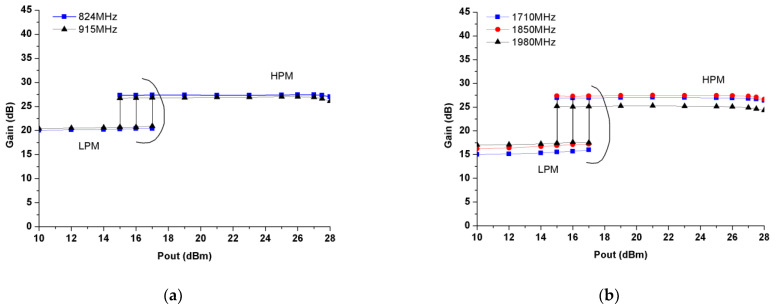
Measured gain in the HPM and LPM: (**a**) LB; (**b**) HB.

The measured power gain as a function of output power (Pout) in both HPM and LPM for the LB (at 824 and 915 MHz) and HB (at 1710, 1850 and 1980 MHz) are plotted in Figure 14a,b, respectively. On the one hand, the LB PA achieves a large-signal power gain of 26.1–27 dB/20.4–20.9 dB with P_1dB_ of >28 dBm/>17 dBm in the HPM/LPM. On the other hand, the HB PA reveals a power gain of 24.3–26.7 dB/15.9–17.5 dB with P_1dB_ of >28 dBm/>17 dBm in the HPM/LPM. Both LB and HB PA blocks exhibit a 6–8 dB superior power gain in the HPM than that in the LPM since the aided-chain amplifiers are disactivated in the LPM.

**Figure 15:**
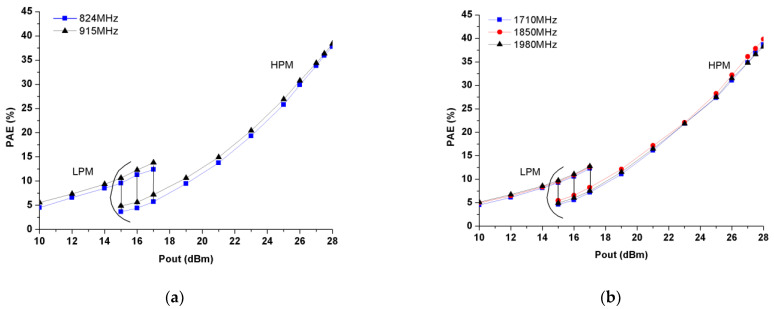
Measured PAE in the HPM and LPM: (**a**) LB; (**b**) HB.

Figure 15a,b present the measured PAE as a function of output power (Pout) in both HPM and LPM for the LB and HB PA blocks, respectively. The PAEs of as high as 38–38.4% at a Pout of 28 dBm in the HPM and of 12.4–13.8% at a Pout of 17 dBm in the LPM are obtained in the LB PA block at 824 and 915 MHz, while the HB block shows PAEs of 38.2–39.9% at a Pout of 28 dBm in the HPM and of 12.3–12.8% at a Pout of 17 dBm in the LPM at 1710, 1850 and 1980 MHz. Since only 4–5% PAE are achieved at the Pout of 17 dBm in the HPM, both LB and HB PA blocks exhibit approximately 7–8% better PAEs in the LPM than in the HPM. Thus, the measurement results demonstrate that the dual power mode architecture can effectively improve the efficiency of the proposed PAM at power back off.

The main measured RF performances, especially linearity characteristics, of the proposed MMMB PAM are summarized in Table 1. Benefitting from the broadband output matching design, the LB and HB PA blocks show a output third-order intercept point (OIP3) of >40.5/>41.8 dBm, and 2nd-/3rd-order harmonic suppression of <−37/<−54 dBc and <−38/<−50 dBc, respectively, in both power modes. The measured adjacent channel leakage ratios (ACLRs) of the proposed PAM under different modulated signals are compared. Measured with WCMDA mode with 5/10 MHz offset, the LB PA at both 824 and 915 MHz generates ACLR-5/ACLR-10 of <−38.2/<−52 dBc for the HPM under Pout of 28 dBm and <−40/<−59.1 dBc for the LPM under Pout of 17 dBm, while the HB one exhibits ACLR-5/ACLR-10 of <−35.9/<−51.8 and <−36.6/<−52.2 dBc for the HPM and LPM, respectively, at 1710, 1850 and 1980 MHz under the same conditions. Furthermore, the PAM in LTE mode shows ACLR_E_UTRA_ of <−33.5 dBc and ACLR_UTRA_ of <−33.2 dBc for both power modes in both bands.

The performance comparison between this work and previous reported results is listed in Table 2. It can be observed that the proposed hybrid CMOS/HBT/SOI PAM exhibits the comparable performance over other state-of-the-art reported PAMs. Particularly, with the use of the 3D vertical stack MCM package strategy, the proposed PAM covering five frequency bands and operating at two power modes only occupies a 5 × 3.5 mm^2^ area, which achieves the smallest chip size compared to the published work in Table 2. To the best of the authors’ knowledge, this work is the first demonstration of a MMMB PAM using 3D vertical stack MCM package to achieve extremely small size with favorable RF performance.

## 4. Conclusions

A fully integrated MMMB PAM with one bulk CMOS die, one GaAs HBT die and one switch die on a six-layer laminate has been developed for mobile handset applications. On the one hand, by using a parallel dual-chain two-stage PA strategy with broadband matching technique, the PAM is qualified for operating at two power modes for different power levels in both LB and HB, thereby improving back-off efficiency with multiband operation. The implemented PAM delivers sufficient output power and exhibits PAEs as high as 38–39.9% for HPM and 12.4–13.8% for LPM. On the other hand, the ultra-compact 3D vertical stack MCM package structure in which the CMOS die is 3D stacked vertically on the HBT die contributes significantly to chip size reduction. The fabricated PAM covering five frequency bands only occupies a 5 × 3.5 mm^2^ chip size. It is a demonstration that the PAM, with its related design and package techniques, is capable of meeting the stringent requirements of modern mobile communication systems. The proposed wideband high-efficiency PAM can be a new practical solution to the realization of MMMB cellular handsets with smaller chip size.

## Figures and Tables

**Table 1:** Measured performance summary of proposed MMMB PAM.

Band	Freq. (MHz)	Pout (dBm)	Gain (dB)	PAE (%)	OIP3 (dBm)	H2 (dBc)	H3 (dBc)	ACLR-5 ^1^ (dBc)	ACLR-10 ^2^ (dBc)	ACLR_E_UTRA_^3^ (dBc)	ACLR_UTRA_^4^ (dBc)
LB	824	28	27	38	40.6	−38.2	−61	−39.6	−52.8	−34.5	−35
		17	20.4	12.4	42.7	−37.2	−57.4	−40	−60.2	−36.5	−35.5
	915	28	26.1	38.4	40.5	−37.1	−55.8	−38.2	−52	−34.6	−34.6
		17	20.9	13.8	41.8	−38.8	−54	−42.7	−59.1	−37	−35.2
HB	1710	28	26.4	38.7	40.5	−43.3	−59.2	−37.5	−52.3	−33.5	−34.4
		17	15.9	12.3	42.2	−38.7	−54.5	−38.3	−52.2	−35	−35
	1850	28	26.7	39.9	42.8	−45.6	−65.4	−40	−54.1	−34.6	−35.6
		17	17.2	12.6	43.7	−42.3	−51.7	−40.7	−54.2	−36.5	−36.2
	1980	28	24.3	38.2	41.4	−42.2	−50	−35.9	−51.8	−33.5	−33.2
		17	17.5	12.8	41.8	−39.7	−51.1	−36.6	−54.9	−34.6	−34.5

^1^ ACLR for WCDMA mode with 5 MHz offset; ^2^ ACLR for WCDMA mode with 10 MHz offset; ^3^ ACLR for LTE mode with the specification of E-UTRA; ^4^ ACLR for LTE mode with the specification of UTRA.

**Table 2:** Performance comparison between this work and previous reported studies.

Reference			This Work			[27]		[28]			[29]			[30]		
Band	Mode	Freq. (MHz)	824		915	824	915	824		915	824		915	824		915
LB	HPM	Pout (dBm)	28		28	28	28	28		28	33		33	28.2		28.2
		Gain (dB)	27		26.1	28.4	28.6	>26		>27	39.5		37	28.4		28.1
		PAE (%)	38		38.4	39	40.1	40.1–41.1			>35		>35	43		43.2
	LPM	Pout (dBm)	17		17	17	17	16		16	--		--	16		16
		Gain (dB)	20.4		20.9	18.5	16.8	15		>17	--		--	>17		>16
		PAE (%)	12.4		13.8	15.8	14.8	>16		>16	--		--	14.5		14.5
		Freq. (MHz)	1710	1850	1980	1850	1980	1710	1850	1980	1710	1810	1910	1710	1850	1910
HB	HPM	Pout (dBm)	28	28	28	28	28	28	28	28	30.5	30.5	30.5	27.5	28.5	28
		Gain (dB)	26.4	26.7	24.3	31.2	30.7	>23.5	>23.5	>26	35.5	35.5	35	28.2	26.6	27.8
		PAE (%)	38.7	39.9	38.2	34.9	35.1	40.1–41.1			>33	>33	>33	38.5	40.7	39
	LPM	Pout (dBm)	17	17	17	17.5	17.5	16	16	16	--	--	--	16	16	16
		Gain (dB)	15.9	17.2	17.5	19.1	19.1	>17.5	>17.5	>18.5	--	--	--	>17	>17	>17
		PAE (%)	12.3	12.6	12.8	15.4	15.1	>24	>24	>24	--	--	--	14.5	14.5	14.5
Technology			Hybrid CMOS/HBT/SOI			Hybrid CMOS/HBT		HBT			Hybrid CMOS/HBT/SOI			HBT		
Package			3D stack MCM			Planar MCM		Planar MCM			Planar MCM			Planar MCM		
Size (mm^2^)			5 × 3.5			5 × 5.5		5 × 7			5 × 5			5 × 6		

## Data Availability

The presented data in this paper are available on request from the first author.
